# Cytoprotective Role of Edible Seahorse (*Hippocampus abdominalis*)-Derived Peptides in H_2_O_2_-Induced Oxidative Stress in Human Umbilical Vein Endothelial Cells

**DOI:** 10.3390/md19020086

**Published:** 2021-02-03

**Authors:** Yunok Oh, Chang-Bum Ahn, Jae-Young Je

**Affiliations:** 1 Institute of Marine Life Science, Pukyong National University, Busan 48513, Korea; si565@hanmail.net; 2 Division of Food and Nutrition, Chonnam National University, Gwangju 61186, Korea; a321@jnu.ac.kr; 3 Department of Marine-Bio Convergence Science, Pukyong National University, Busan 48547, Korea

**Keywords:** seahorse, cytoprotective peptides, oxidative stress, HO-1, Nrf2, antiapoptosis

## Abstract

Oxidative stress-induced endothelial dysfunction is strongly linked to the pathogenesis of cardiovascular diseases. A previous study revealed that seahorse hydrolysates ameliorated oxidative stress-mediated human umbilical vein endothelial cells (HUVECs) injury. However, the responsible compounds have not yet been identified. This study aimed to identify cytoprotective peptides and to investigate the molecular mechanism underlying the cytoprotective role in H_2_O_2_-induced HUVECs injury. After purification by gel filtration and HPLC, two peptides were sequenced by liquid chromatography-tandem mass spectrometry as HGSH (436.43 Da) and KGPSW (573.65 Da). The synthesized peptides and their combination (1:1 ratio) showed significant HUVECs protection effect at 100 μg/mL against H_2_O_2_-induced oxidative damage via significantly reducing intracellular reactive oxygen species (ROS). Two peptides and their combination treatment resulted in the increased heme oxygenase-1 (HO-1), a phase II detoxifying enzyme, through the activation of nuclear transcription factor-erythroid 2-related factor (Nrf2). Additionally, cell cycle and nuclear staining analysis revealed that two peptides and their combination significantly protected H_2_O_2_-induced cell death through antiapoptotic action. Two peptides and their combination treatment led to inhibit the expression of proapoptotic Bax, the release of cytochrome C into the cytosol, the activation of caspase 3 by H_2_O_2_ treatment in HUVECs, whereas antiapoptotic Bcl-2 expression was increased with concomitant downregulation of Bax/Bcl-2 ratio. Taken together, these results suggest that seahorse-derived peptides may be a promising agent for oxidative stress-related cardiovascular diseases.

## 1. Introduction

An imbalance between reactive oxygen species (ROS) production and antioxidant defense is called “oxidative stress”. Atherosclerosis is a chronic vascular disorder caused by oxidative stress that causes lipid oxidation of cholesterols and lipoproteins [1]. Accumulated these events result in endothelial cell injury that plays a pivotal role in the pathogenesis of atherosclerosis. Endothelial dysfunction caused by oxidative stress is an early marker for atherosclerosis, which is mediated by decreasing vasoprotective effects of the endothelium, including nitric oxide production for vasodilation and inhibiting inflammation [2,3]. Several studies have clearly shown that endothelial dysfunction is associated with cardiovascular diseases (CVD) [3,4,5]. Pathogenesis mechanism in atherosclerosis by oxidative stress is linked to increases in vascular endothelial cell apoptosis and mitochondria dysfunction [1,6]. Since oxidative stress is considered a major risk for atherosclerosis, it is important for ameliorating oxidative stress in the body through antioxidant intervention.

The seahorse (*Hippocampus abdominalis*) is a marine teleost fish, and the dried seahorse is used for traditional Chinese medicine. Several biological effects have been shown in association with antioxidant, neuroprotective, anti-arthritis, and antithrombosis from seahorses (*H. kuda* Bleeler and *H. trimaculatus* Leach) [7,8,9,10]. Recently, edible seahorse (*H*. *abdominalis*) hydrolysates by enzymatic hydrolysis have been reported to have versatile biological activities, including angiotensin-converting enzyme inhibitory activity, anti-fatigue effect, and cell differentiation [11,12,13]. In addition, this hydrolysate by pepsin and Alcalase^®^ have also been reported to exert antioxidant activity in oxidative stress-mediated HUVECs and zebrafish models [14,15]. However, bioactive peptides (BAPs) that are responsible for HUVECs protection have not been identified yet, as well as the exact molecular mechanism. Several lines of evidence have demonstrated that marine organisms are a good source for BAPs with health promotion [16,17,18]. In particular, BAPs are extensively developed for natural antioxidants to use functional food ingredients and/or nutraceuticals. It is generally produced by enzymatic hydrolysis and further purified and identified from marine food proteins such as sea squirt, ark shell, krill, blue mussel, blood cockle [19,20,21,22,23]. Thus, the aims of this study were to purify and identify BAPs that are responsible for HUVECs protection from edible seahorse (*H*. *abdominalis*) hydrolysates and to elucidate the mechanism of action underlying the cytoprotection of the isolated BAPs in H_2_O_2_-induced HUVECs injury through measuring antioxidant and antiapoptotic activity.

## 2. Results

### 2.1. Purification and Identification of BAPs from Edible Seahorse Hydrolysates by Alcalase^®^

**Figure 1:**
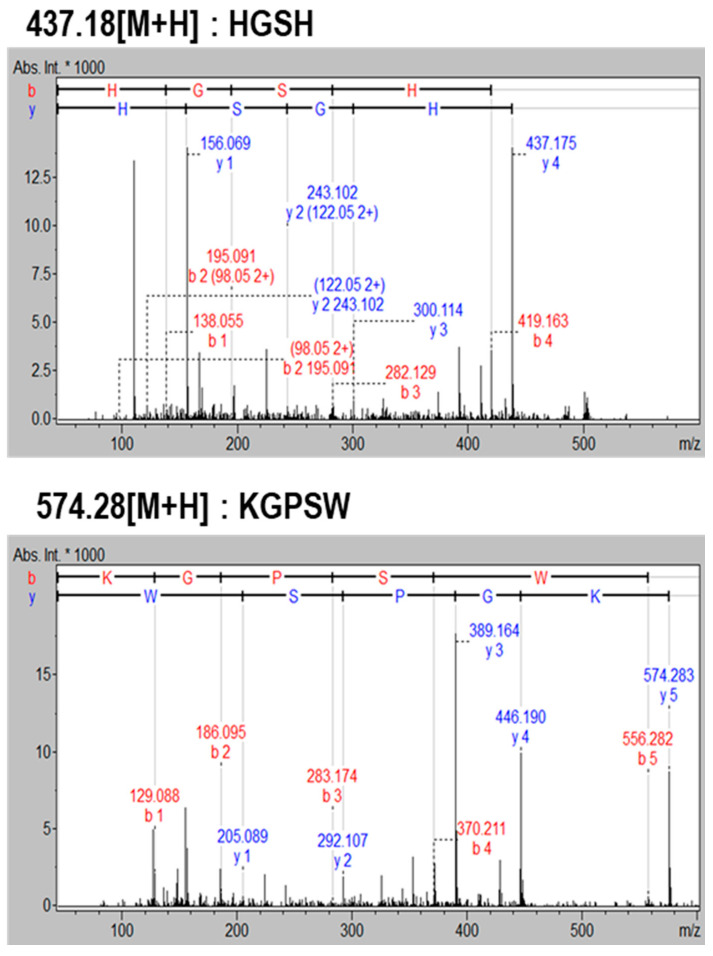
Identification of cytoprotective peptides from seahorse hydrolysates using LC–MS/MS.

Initially, Sephadex G-25 gel filtration chromatography was employed to separate BAPs from edible seahorse hydrolysates by Alcalase^®,^ and the separated BAPs fraction was subjected to HUVECs protection activity against H_2_O_2_-induced injury. Four fractions (F1–4) were obtained, and among four fractions, the F3 fraction at 0.5 mg/mL exhibited higher HUVEC protection than F1, F2, and F4 fractions (Supplementary Figure S1A,B). To the next step, F3 was subjected to HPLC equipped with a C_18_ column and was separated by four fractions (H1–4). Among H1–4 fractions, H2 and H3 showed higher HUVEC protection activity than H1 and H4 fractions (Supplementary Figure S1C,D). Thus, H2 and H3 fractions were subjected to BAPs identification by Q-TOF LC–MS/MS. However, BAPs identification in the H2 fraction was failed, but two BAPs were identified from the H3 fraction. The MS/MS spectra of two BAPs are shown in Figure 1. The BAPs sequence was found to be HGSH (436.43 Da) and KGPSW (573.65 Da). The BAPs content in the final stage of purification by HPLC is around 1.1%, calculated by MS intensity. Two BAPs were chemically synthesized to evaluate the cytoprotective effect, and the purities of BAPs were above 96%.

### 2.2. Two BAPs Protect H_2_O_2_-Induced HUVECs Damage

**Figure 2:**
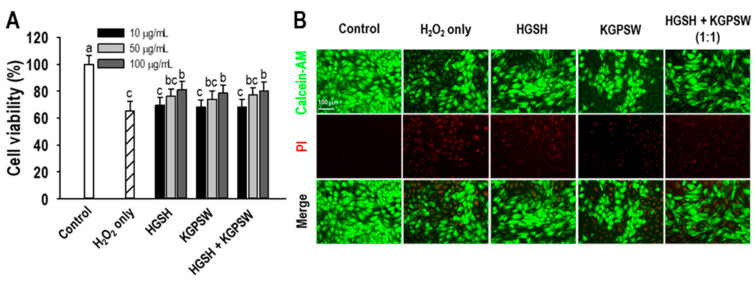
(**A**) Effect of HGSH, KGPSW and their combination (1:1) on oxidative stress-mediated HUVEC injury. (**B**) Live-dead cell assay by calcein-AM/PI double-staining of treatment with 0.1 mg/mL of HGSH, KGPSW, and their combination. Cells were treated with peptide for 2 h, followed by exposure to 600 μM H_2_O_2_ and further incubation for 24 h. Values are presented as means ± SD (*n* = 4). ^a–c^ Different letters mean significant difference at *p* < 0.05.

Since two BAPs were identified in the same fraction, this study was designed to evaluate the cytoprotective activity following treatment of HGSH, KGPSW and 1:1 ratio of HGSH and KGPSW. This is the reason for checking the synergy effect of two BAPs. First, cytotoxicity of two BAPs against HUVECs was determined prior to evaluating cytoprotective activity, and there is no effect on HUVECs viability in the tested concentration (data not shown). Then, this study was carried out the cytoprotection activity in the H_2_O_2_-induced HUVECs injury. A 600 μM H_2_O_2_ treatment, which was a previously determined concentration, resulted in a significant decrease in HUVECs viability (65.43%) compared to that of control (without treatment) by 3-(4,5-dimethylthiazol-2-yl)-2,5-diphenyltetrazolium bromide (MTT) assay (Figure 2A). However, pretreatment of two BAPs and their combination in the H_2_O_2_-induced HUVECs injury increased the HUVECs viability up to 81.02% (HGSH), 78.55% (KGPSW), and 80.05% (1:1 combination) at 100 μg/mL. However, there was no synergic effect in the combination of HGSH and KGPSW on cytoprotective activity. For further confirmation, live/dead cell assay by calcein-AM/PI staining was conducted, and the result showed that H_2_O_2_ treatment only increased the level of PI stained HUVECs, but this was decreased by pretreatment of two BAPs and their combination (Figure 2B). The calcein-AM/PI staining assay results were similar to those of the MTT assay. We also tested 2,2-diphenyl-1-picrylhydrazyl (DPPH) radical scavenging activity of two BAPs at 100 μg/mL, and the results showed that two BAPs did not scavenge DPPH radical (data not shown).

### 2.3. Two BAPs Inhibit Intracellular ROS Generation and Activate HO-1/Nrf2 Pathway

**Figure 3:**
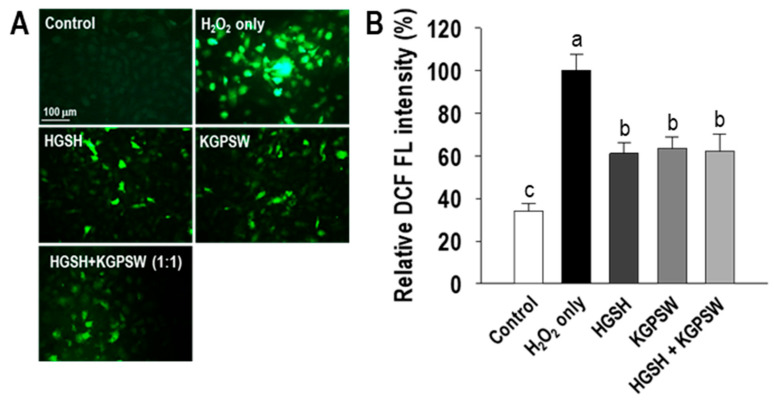
Determination of intracellular ROS generation. (**A**) Representative image and (**B**) quantification of fluorescence intensity. Cells were treated with 0.1 mg/mL of two cytoprotective peptides (HGSH, KGPSW), and its combination (1:1) for 2 h, followed by exposure to 600 μM H_2_O_2_ and further incubation for 24 h. Values are presented as means ± SD (*n* = 4). ^a–c^ Different letters mean significant difference at *p* < 0.05.

To examine whether the cytoprotective activity of two BAPs in the H_2_O_2_-induced HUVECs damage was related to antioxidant ability, the intracellular ROS generation in HUVECs was determined by a DCFH-DA fluorescent probe. High-level intracellular ROS in cells can generate the fluorescent DCF. As shown in Figure 3A, the DCF fluorescent signal in the control group was not detectable, but a high level of the DCF fluorescent signal in the H_2_O_2_ treatment was observed, indicating a high amount of intracellular ROS in HUVECs. However, pretreatment with HGSH, KGPSW and their combination in the H_2_O_2_-induced HUVECs injury considerably reduced the DCF fluorescent signal compared to that of the H_2_O_2_ treatment group. The result of relatively calculated DCF fluorescent intensity showed that HGSH, KGPSW and their combination significantly inhibited intracellular ROS generation by around 40% compared to that of the H_2_O_2_ treatment group.

**Figure 4:**
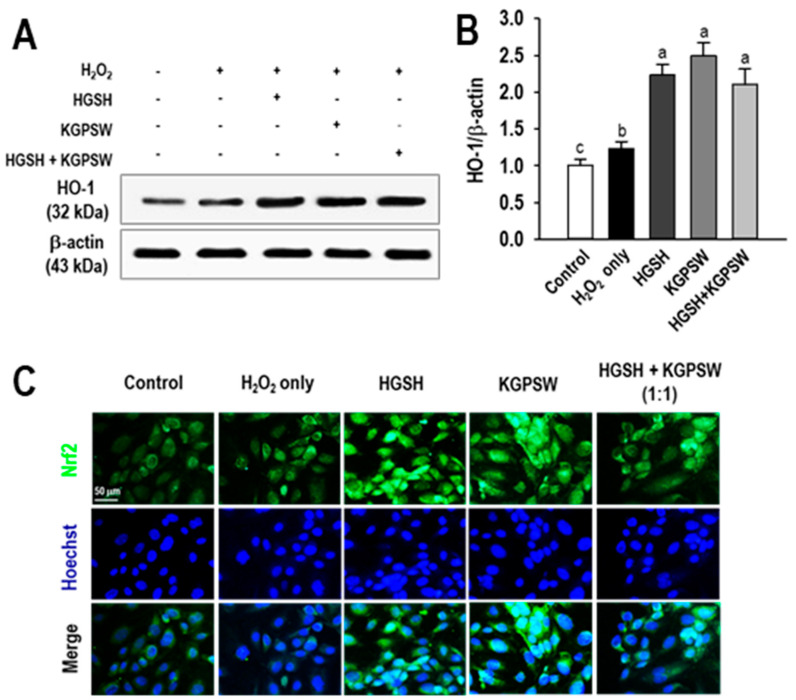
Effect of HGSH, KGPSW and their combination on heme oxygenase-1 (HO-1) expression and nuclear accumulation of nuclear transcription factor-erythroid 2-related factor (Nrf2) in oxidative stress-mediated human umbilical vein endothelial cells (HUVEC) injury. (**A**) Western blot analysis of HO-1 and (**B**) quantification of HO-1 expression and (**C**) immunofluorescence staining of Nrf2 nuclear accumulation. Cells were treated with 0.1 mg/mL of HGSH, KGPSW, and their combination (1:1) for 2 h, followed by exposure to 600 μM H_2_O_2_ and further incubation for 24 h (HO-1 analysis) or 2 h (Nrf2 analysis). Values are presented as means ± SD (*n* = 3). ^a–c^ Different letters mean significant difference at *p* < 0.05.

To confirm the inhibitory effect of two BAPs on intracellular ROS generation, the activations of a key cytoprotective enzyme and a transcription factor were investigated. Nrf2, a transcription factor, is involved in the expression of antioxidants and phase II detoxifying enzymes in response to oxidative stress, which is related to cellular protection and longevity [24]. As shown in Figure 4A,B, the induction of HO-1, a key cytoprotective enzyme that is regulated by Nrf2, in the H_2_O_2_ treatment group was slightly increased compared to the control group in response to H_2_O_2_ addition. However, this induction was not sufficient to protect HUVECs. However, pretreatment of HGSH, KGPSW, and their combination in the H_2_O_2_-induced HUVECs injury significantly increased the expression of HO-1 compared to that of the H_2_O_2_ treatment group, thereby exhibiting the cytoprotection effect. To further confirm HO-1 induction by two BAPs and their combination, Nrf2 nuclear translocation was examined by immunofluorescence staining assay in the H_2_O_2_-induced HUVECs injury. As shown in Figure 4C, immunofluorescence staining showed that most of Nrf2 were detectable in the cytoplasm, whereas Nrf2 in the nucleus was low in the control group. In the H_2_O_2_ treatment group, Nrf2-reactive fluorescent signals were detectable in the nucleus of HUVECs compared to that of the control group. Notably, Nrf2 expressions in both the cytoplasm and the nucleus were remarkably detectable in two BAPs and their combination treating HUVECs, indicating HO-1 induction by Nrf2 nuclear translocation.

### 2.4. Antiapoptotic Action of Two BAPs and Their Combination in H_2_O_2_-Induced HUVECs Damage

**Figure 5:**
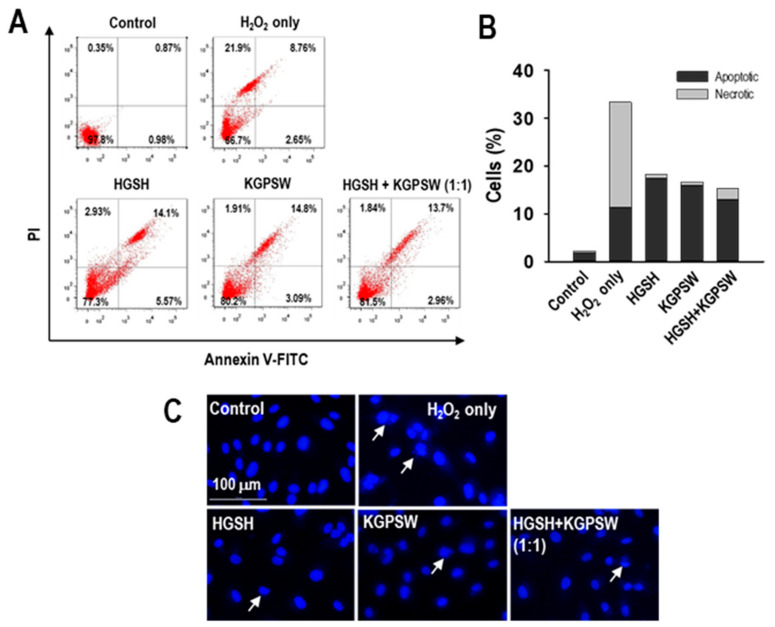
Determination of the inhibitory effect of HGSH, KGPSW, and their combination on H_2_O_2_-induced apoptosis in HUVEC cells. (**A**) Representative quadrant dot plots of apoptosis and (**B**) quantification of apoptotic and necrotic percentages by flow cytometer analysis with annexin V/ propidium iodide (PI) double staining. (**C**) Cell morphological changes using Hoechst 33342 nuclear staining under a fluorescence microscope (white arrows indicate apoptotic cells). Cells were treated with 0.1 mg/mL of HGSH, KGPSW, and their combination (1:1) for 2 h, followed by exposure to 600 μM H_2_O_2_ and further incubation for 24 h. Experiments were performed in triplicate.

It has been reported that H_2_O_2_ causes cell damage leading to cell death by processes of apoptosis and/or necrosis [25]. Thus, annexin V-FITC/PI double and Hoechst 33342 staining assays were performed to detect apoptosis and/or necrosis. As shown in Figure 5A,B, the control cells were double negative, and most of the cells (97.80%) were in the lower left quadrant, whereas the H_2_O_2_-treated HUVECs showed 11.41% apoptotic cells and 21.90% necrotic cells. Although pretreatment of two BAPs and their combination decreased cell death, the apoptotic cells were higher than that of the H_2_O_2_-treated HUVECs, but necrotic cells were lower. Nuclear staining results showed that the control HUVECs showed round and intact nuclei, but nuclear morphology was changed to a shrunk, segregated and punctuated nucleus in response to H_2_O_2_ treatment (Figure 5C). Pretreatment with two BAPs and their combination in the H_2_O_2_-induced HUVECs injury reduced morphological changes.

**Figure 6:**
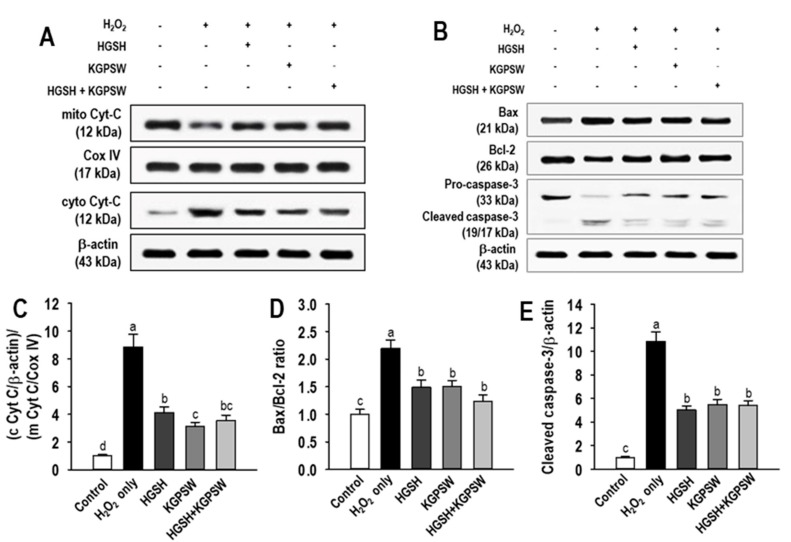
Effect of HGSH, KGPSW, and their combination on apoptosis-related proteins in H_2_O_2_-mediated HUVEC injury by Western blot analysis. (**A**) Cytochrome C (Cyt-C) release from the mitochondria into the cytosol, (**B**) the expression of Bax, Bcl-2, and activated caspase-3 (procaspase-3/cleaved caspase-3), and quantification analysis from the Western blot results of (**C**) Cyt C release in mitochondria and cytoplasmic fractions, (**D**) Bax/Bcl-2 ratio, and (**E**) caspase-3 activation using cleaved caspase-3/β-actin. Cells were treated with 0.1 mg/mL of HGSH, KGPSW, and their combination (1:1) for 2 h, followed by exposure to 600 μM H_2_O_2_ and further incubation for 24 h. Cox IV and β-actin are shown as protein loading controls. Results are representative of three separate experiments. Values are presented as means ± SD (*n* = 3). ^a–c^ Different letters mean significant difference at *p* < 0.05.

It is well-known that stress signals such as the high concentration of H_2_O_2_ induced apoptosis by the intrinsic pathway that leads to mitochondrial cytochrome C release dependent cell death [26]. Thus, the effect of two BAPs and their combination on the intrinsic pathway in H_2_O_2_-induced HUVECs injury was investigated. As shown in Figure 6A,C, the H_2_O_2_-treated HUVECs remarkably increased the release of cytochrome C (Cyt-C) from the mitochondria into the cytosol compared to the control group, whereas pretreatment with two BAPs and their combination significantly reduced Cyt-C release into the cytosol in the H_2_O_2_-induced HUVECs injury. Next, we examined the expression of Bax, a proapoptotic protein and Bcl-2, an antiapoptotic protein, because the release of Cyt-C is governed by the relative expression of Bax and Bcl-2 proteins [25]. As shown in Figure 6B,D, Bax expression was increased by treatment with H_2_O_2_ alone, whereas Bcl-2 expression was decreased compared to those of the control group, resulting in an increase in the Bax/Bcl-2 ratio, indicating activation of the intrinsic pathway. However, the increased Bax/Bcl-2 ratio was decreased by pretreatment with two BAPs and their combination. The effect of two BAPs and their combination on activation of caspase-3 was finally investigated. As shown in Figure 6B,E, the active form of caspase-3, the cleaved caspase-3, was significantly detected in the H_2_O_2_-treated HUVECs, indicating apoptosis. However, the cleaved caspase-3 was reduced by pretreatment with two BAPs and their combination.

## 3. Discussion

Although there are specifically pharmacological actions of seahorses, it is scantily reported with reference to bioactive peptides with cytoprotective effects from seahorses. In this study, two cytoprotective peptides (HGSH and KGPSW) from edible seahorse (*H*. *abdominalis*) were purified, identified and demonstrated the cytoprotective role of HGSH and KGPSW in oxidative stress-mediated HUVECs injury. To the best of our knowledge, this is the first report about the cytoprotective peptides from edible seahorse (*H*. *abdominalis*).

The main protective strategy to reduce oxidative damage is to provide exogenous antioxidant molecules and to induce an antioxidant defense system in cells. ROS are highly reactive substances that could react with biomacromolecules, and overproduced ROS are considered as the main factor for the onset of diseases. Moreover, endothelial dysfunction and apoptosis induced by oxidative damage are involved in the pathogenesis of CVD [27]. Therefore, ameliorating oxidative damage in endothelial cells may be a crucial step in the prevention of CVD. Accordingly, HUVECs with oxidative stress conditions are widely used to evaluate CVD prevention potential [28,29,30]. Moreover, most studies used H_2_O_2_ as an oxidative stress marker, which is an important metabolite in the redox metabolism of the cells, and HUVECs will suffer from the cytotoxicity of H_2_O_2_. The present study is also found that the HUVECs viability after exposure to H_2_O_2_ was significantly decreased, but pretreatment with HGSH, KGPSW, and their combination significantly inhibited H_2_O_2_-mediated HUVECs injury. Mechanisms underlying the cytotoxicity by H_2_O_2_ may include the depletion of the antioxidant defense system and the activation of apoptosis-related signals [28]. Thus, we investigated whether HGSH, KGPSW, and their combined influence cellular antioxidant capacity and apoptosis-related signaling pathway. As expected, H_2_O_2_ treatment increased intracellular ROS generation in HUVECs, and this increase was decreased by two BAPs and their combination. To verify the inhibiting activity for intracellular ROS generation, we focused on the activation of HO-1/Nrf2 signaling. HO-1 is a well-known cytoprotective enzyme and catalyzes heme degradation into CO, ferritin, and biliverdin, which is further converted to bilirubin by biliverdin reductase [31]. Biliverdin/bilirubin are potent antioxidants and provide cellular protective effects through scavenging ROS [32]. CO exerts anti-inflammatory and antiapoptotic action in endothelial cells [31,33]. Therefore, the induction of antioxidant defense system by exogenous sources is a promising intervention to reduce oxidative damage. Indeed, two BAPs and their combination resulted in the increase in HO-1 expression in the H_2_O_2_-induced HUVECs injury, which is thought to increase HUVEC survival by scavenging intracellular ROS. Nuclear translocation of Nrf2, a transcription factor, is required to activate an endogenous defense system against oxidative stress. It can activate antioxidant and phase II detoxifying enzymes such as HO-1 that quench ROS. Under physiological conditions, it binds to Kelch-like ECH-associated protein 1 that promotes ubiquitination of Nrf2, leading to the cytosolic sequestration followed by degradation [34]. However, Nrf2 translocates into the nucleus, followed by binding to the antioxidant-related elements in response to oxidative stress, which leads to upregulate antioxidant and cytoprotective enzymes [35]. In this study, immunofluorescence staining results demonstrated that Nrf2 translocation and accumulation into the nucleus by pretreatment with two BAPs and their combination was augmented, thus facilitated the expression of HO-1. These results are similar to the results of recent studies [29,36,37,38,39]. We also measured the direct antioxidant effect of two BAPs using DPPH radical scavenging assay to check that two BAPs showed the cytoprotective effect through HO-1/Nrf2 signaling. Two BAPs did not scavenge DPPH radicals. This result suggested that two BAPs exert the cytoprotective effect in H_2_O_2_-induced HUVECs injury through upregulating cellular HO-1 activity. Some BAPs that protect HUVECs from oxidative stress has been reported, and most are hydrolysates. Two peptides with the sequence of Lys–His–Asn–Arg–Gly–Asp–Glu–Phe from rice bran protein and Phe–Pro–Tyr–Leu–Arg–His from miiuy croaker protein showed the cytoprotective effect with the increased cell viability in H_2_O_2_-induced HUVECs injury [40,41]. Comparing the sequence of two BAPs and those in the literature, there are antioxidant amino acids such as Tyr, His, Pro, Phe, Trp, and Lys (*N*-terminal), but there is no similarity in the sequence.

Next, we assessed the antiapoptotic effect of HGSH, KGPSW, and their combination to further verify the cytoprotective role in the H_2_O_2_-induced HUVECs injury because of H_2_O_2_ results in both apoptosis and necrosis [25]. In the H_2_O_2_-treated HUVECs, both apoptotic and necrotic cells were observed, whereas HGSH, KGPSW, and their combination treatment decreased cell death through decreasing necrotic cells, but not apoptotic cells. This can be assumed that apoptotic cells were initially detected by treatment of H_2_O_2_ alone, then most apoptotic cells were finally detected as necrotic cells in our model. To prove the antiapoptotic effects of HGSH, KGPSW, and their combination, changes in the mitochondrial pathway was investigated because of H_2_O_2_ triggers apoptosis through the intrinsic pathway [26]. Upon exposure of H_2_O_2_, it leads to the change in the ratio of Bax/Bcl-2 expression, which results in the increased permeability of the mitochondrial membrane with Cyt-C release into the cytosol. The increased Cyt-C concentration in the cytosol leads to the activation of caspase-9, which in turn activates caspase-3, a form of cleaved caspase-3, which is the final executioner of apoptosis [26]. In line with the above pathway, a number of studies have demonstrated the antiapoptotic effects of BAPs, such as rice bran peptides, *Mytilus coruscus* peptides, and blue mussel peptides, in H_2_O_2_-induced HUVECs injury [29,36,41]. In this study, the levels of the ratio of Bax/Bcl-2, the release of Cyt-c into the cytosol, and the cleaved caspase-3 were increased in the H_2_O_2_-induced HUVECs injury, indicating apoptosis through the activation of the mitochondrial pathway. These observations are similar to those in the literature above. Pretreatment of HGSH, KGPSW, and their combination resulted in the reversal of the apoptosis-related events in the H_2_O_2_-induced HUVECs injury. These results suggest that HGSH, KGPSW, and their combination could protect HUVECs from H_2_O_2_-mediated oxidative damage by enhancement of antioxidant defense capacity and suppressing apoptosis.

## 4. Material and Methods

### 4.1. Materials

Edible seahorse (*H*. *abdominalia*), a farmed seahorse, was purchased from the Center for Ornamental, Reef and Aquariums (Jeju, Korea), and this is originated from New Zealand. 2′7′-dichlorofluorescin diacetate (DCFH-DA), H_2_O_2_, and MTT were purchased from Sigma-Aldrich Chemical Co. (St. Louis, MO, USA). Primary HUVECs were purchased from the American Type Culture Collection (ATCC) (Rockville, MD, USA). EGM^TM^-2 endothelial growth medium-2 BulletKit was obtained from Lonza (Walkersville, MD, USA). All commercial chemicals were used without further purification.

### 4.2. Preparation of Edible Seahorse Hydrolysates by Alcalase

Edible seahorse hydrolysates by Alcalase^®^ 2.4 L (Novozyme, Bagsvaerd, Denmark) were prepared according to our previous method [15]. Briefly, freeze-dried seahorse powder (10 g) in 100 mL distilled water was mixed with Alcalase^®^ 2.4 L (0.1 g) at 50 °C and pH 8.0 followed by incubation for 8 h. The reaction mixture was heated at 100 °C for 10 min, and the supernatant was collected by centrifugation (5000 rpm, 20 min) followed by freeze-drying and storing at −20 °C until use.

### 4.3. Purification and Identification of Cytoprotective Peptides from Edible Seahorse Hydrolysates

Peptides with potent cytoprotective effect were purified using a Sephadex G-25 gel filtration followed by HPLC (Dionex Ultimate 3000, Thermo Scientific, Pittsburgh, PA, USA) equipped with a C_18_ column (Hypersil Gold, 250 × 10 mm, 5 µm, Thermo Scientific). Briefly, the edible seahorse hydrolysates were loaded and eluted at a flow rate of 1.0 mL/min on a Sephadex G-25 column (3.0 × 90 cm), and the eluate was collected every 5 min. The active fraction showing cytoprotective activity was further separated on the C_18_ column at a flow rate of 2.5 mL/min. The elution was done using a linear gradient of acetonitrile (0% in 5 min, 0–50% in 25 min, 100% in 25–27.5 min, and 0% in 27.5–30 min) containing 0.05% trifluoroacetic acid.

Peptide identification was done according to our previous report [20] with an ultra-high-resolution Q-TOF LC–MS/MS coupled with an ESI source (maXis-HD^TM^, Bruker Daltonics, Bremen, Germany). The de novo peptide sequencing program using MS/MS spectrometry (Biotools 3.2 software, Bruker Daltonics) was used for peptide sequencing.

### 4.4. Peptide Synthesis

The identified peptides were chemically synthesized and supplied by Biostem (Ansan, Korea). The synthesized peptides were above 97% purity (HPLC-MS/MS, Shimadzu LC-20A, Kyoto, Japan).

### 4.5. Cell Culture and Peptide Treatment

Primary HUVECs were cultured in EGM^TM^-2 endothelial growth medium-2 BulletKit in a humidified incubator at 37 °C and 5% CO_2_. HUVECs in passages 3 to 5 were used in the study.

After confluency, HUVECs were treated with peptides for 2 h prior to exposure of 600 μM H_2_O_2_ and then incubated for 24 h or 2 h.

### 4.6. Cell Viability Assessment

HUVECs were seeded into a 96-well plate at a density of 1 × 10^4^ cells/well. After treatment, 0.5 mg/mL of MTT solution was added and incubated for 4 h. The formazan crystals were dissolved in DMSO followed by checking absorbance at 540 nm (GENios microplate reader, GENios, TECAN, Männedorf, Switzerland).

For live and dead cell assay, HUVECs were seeded into a 24-well plate at a density of 2 × 10^4^ cells/well. After treatment, HUVECs were rinsed three times with phosphate-buffered saline (PBS) and stained with 2.5 μM calcein-AM and 5 μM propidium iodide (PI) staining reagent (BD Bioscience, San Jose, CA, USA) at 37 °C for 30 min. Stained cells were visualized under a fluorescence microscope (Leica DMI 6000B, Wetzlar, Germany).

### 4.7. DPPH Radical Scavenging Assay

DPPH radical scavenging activity of two BAPs were measured according to the method described by Ahn et al. [42]. Briefly, a 100 μL of DPPH working solution (0.15 mM in ethanol) was mixed with 100 μL of two BAPs and then allowed in a dark room for 30 min. Absorbance was measured at 517 nm (GENios, TECAN).

### 4.8. Intracellular ROS Determination

HUVECs were seeded into a 96-well black plate or 24-well plate. After treatment, 20 μM DCFH-DA in HBSS was added into HUVECs followed by incubation for 20 min at 37 °C. Intracellular ROS was determined by measuring fluorescence intensity at excitation and emission of 485 and 528 nm (GENios microplate reader and fluorescence microscope).

### 4.9. Annexin V-FITC/PI Double Staining

The percentage of apoptosis and necrosis HUVECs was measured by flow cytometry (*FACS*Calibur system, BD Biosciences, San Jose, CA, USA) with an annexin V-FITC apoptosis detection kit (BD Pharmingen^TM^, San Jose, CA, USA) according to the manufacturer’s protocol. After treatment, the cells were harvested, washed twice with PBS, and resuspended in binding buffer. A 10 μg/mL of annexin-V FITC/PI double staining solution was added into the cells and then incubated for 15 min at room temperature in the dark. The cells were diluted with binding buffer followed by flow cytometry analysis.

### 4.10. Hoechst 33342 Staining Analysis

Hoechst 33342 staining was conducted to detect the alteration of the nuclear morphology. After treatment, the cells were fixed with ice-cold ethanol for 20 min and then stained with a 10 μM Hoechst 33342 for 20 min at room temperature. The nuclear morphology was observed under a fluorescence microscope.

### 4.11. Western Blot Analysis

Total proteins were extracted with a RIPA buffer (Sigma Chemical Co., St. Louis, MO, USA). The mitochondria and cytosol fractions were extracted using a mitochondria isolation kit (Abcam, Cambridge, MA, USA) according to the manufacturer’s protocol. Twenty-five μg of proteins were separated with 10–12% SDS–PAGE and transferred into nitrocellulose membranes. The membranes were blocked with 5% skim milk for 1 h followed by probing specific antibodies (HO-1, 1:200 dilution; Bax, 1:200 dilution; Bcl-2, 1:200 dilution, cytochrome C, 1:200 dilution; β-actin, 1:500 dilution; Cox IV, 1:500 dilution from Santa Cruz Biotechnology, Santa Cruz, CA, USA; caspase-3, 1:500 dilution from Cell Signaling Technology, Beverly, MA, USA) at 4 °C for overnight. Horseradish peroxidase-conjugated secondary antibody was treated for 2 h. The bands were visualized using a chemiluminescence ECL assay kit (Life Technologies, Seoul, Korea) and were imaged on Davinch-Chemi Imager^TM^ (CAS-400SM, Core Bio, Seoul, Korea).

### 4.12. Immunofluorescence Staining of Nrf2 Nuclear Translocation

Nuclear translocation of Nrf2 was examined by immunofluorescence staining. HUVECs were fixed with 3.7% paraformaldehyde in PBS for 15 min followed by treating permeabilization buffer (0.1% Triton X-100 in PBS) for 10 min. HUVECs were incubated with blocking solution (2% bovine serum albumin) for 30 min at room temperature. After washing with PBS, HUVECs were treated with anti-Nrf2 antibody (1:200 dilution, Santa Cruz Biotechnology) at 4 °C for overnight. The secondary antibody labeled with Alexa Fluor^®^ 488 (1:500 dilution, Santa Cruz Biotechnology, Dallas, TX, USA) was treated for 1 h. Nuclei were counterstained with 2 μg/mL Hoechst 33342 for 10 min. The stained HUVECs were visualized using a fluorescence microscope.

### 4.13. Statistical Analysis

Three separate experiments were performed. Data are reported as means ± standard deviation (SD). Statistical analysis by one-way ANOVA was carried out using SigmaPlot^®^ (Systat Software Inc., San Jose, CA, USA). Significant differences were accepted as a *p*-value < 0.05.

## 5. Conclusions

Two BAPs, HGSH and KGPSW, were purified and identified from edible seahorse hydrolysate, and this study demonstrated the cytoprotective role of HGSH and KGPSW in the H_2_O_2_-mediated HUVEC injury. Two BAPs enhanced cellular antioxidant defense capacity and inhibited H_2_O_2_-mediated HUVECs apoptosis through the downregulation of the mitochondrial pathway. However, the present study only demonstrated the cytoprotective role of two BAPs in vitro; thus, further studies are needed to validate their efficacy in vivo in oxidative stress-mediated HUVEC injury.

## Data Availability

Data is contained within the article or supplementary material.

## Supplementary Materials

The following are available online at https://www.mdpi.com/1660-3397/19/2/86/s1, Figure S1: Purification of cytoprotective peptides.

## References

[1] Sun G.-B., Qin M., Ye J.-X., Meng X.-B., Wang M., Luo Y., Li Z.-Y., Wang H.-W., Sun X.-B. (2013). Inhibitory effects of myricitrin on oxidative stress-induced endothelial damage and early atherosclerosis in ApoE−/− mice. Toxicol. Appl. Pharmacol..

[2] Kao C.-L., Chen L.-K., Chang Y.-L., Yung M.-C., Hsu C.-C., Chen Y.-C., Lo W.-L., Chen S.-J., Ku H.-H., Hwang S.-J. (2010). Resveratrol protects human endothelium from H_2_O_2_-induced oxidative stress and senescence via SirT1 activation. J. Atheroscler. Thromb..

[3] Davignon J., Ganz P. (2004). Role of endothelial dysfunction in atherosclerosis. Circulation.

[4] Higashi Y., Maruhashi T., Noma K., Kihara Y. (2014). Oxidative stress and endothelial dysfunction: Clinical evidence and therapeutic implications. Trends Cardiovasc. Med..

[5] Zhong X.-F., Huang G.-D., Luo T., Deng Z.-Y., Hu J.-N. (2012). Protective effect of rhein against oxidative stress-related endothelial cell injury. Mol. Med. Rep..

[6] Madamanchi N.R., Runge M.S. (2007). Mitochondrial dysfunction in atherosclerosis. Circ. Res..

[7] Ryu B., Qian Z.-J., Kim S.-K. (2010). SHP-1, a novel peptide isolated from seahorse inhibits collagen release through the suppression of collagenases 1 and 3, nitric oxide products regulated by NF-κB/p38 kinase. Peptides.

[8] Xu D., Xu S. (1997). The extract from *Hippocampus trimaculatus* Leach: Its effect of antithrombosis on rats and ingredients analysis. Chin. J. Mar. Drugs.

[9] Ryu B., Qian Z.-J., Kim S.-K. (2010). Purification of a peptide from seahorse, that inhibits TPA-induced MMP, iNOS and COX-2 expression through MAPK and NF-κB activation, and induces human osteoblastic and chondrocytic differentiation. Chem.-Biol. Interact..

[10] Himaya S., Ryu B., Qian Z.-J., Kim S.-K. (2012). Paeonol from *Hippocampus kuda* Bleeler suppressed the neuro-inflammatory responses in vitro via NF-κB and MAPK signaling pathways. Toxicol. In Vitro.

[11] Zhang Y., Ryu B., Cui Y., Li C., Zhou C., Hong P., Lee B., Qian Z.-J. (2019). A peptide isolated from *Hippocampus abdominalis* improves exercise performance and exerts anti-fatigue effects via AMPK/PGC-1α pathway in mice. J. Funct. Foods.

[12] Je J.-G., Kim H.-S., Lee H.-G., Oh J.-Y., Lu Y.A., Wang L., Rho S., Jeon Y.-J. (2020). Low-molecular weight peptides isolated from seahorse (*Hippocampus abdominalis*) improve vasodilation via inhibition of angiotensin-converting enzyme in vivo and in vitro. Process Biochem..

[13] Muthuramalingam K., Kim S.-Y., Kim Y., Kim H.-S., Jeon Y.-J., Cho M. (2019). Bigbelly seahorse (*Hippocampus abdominalis*)-derived peptides enhance skeletal muscle differentiation and endurance performance via activated P38MAPK/AKT signalling pathway: An in vitro and in vivo analysis. J. Funct. Foods.

[14] Kim H.-S., Shin B.-O., Kim S.-Y., Wang L., Lee W., Kim Y.T., Rho S., Cho M., Jeon Y.-J. (2016). Antioxidant activity of pepsin hydrolysate derived from edible *Hippocampus abdominalis* in vitro and in Zebrafish models. Korean J. Fish Aquat. Sci..

[15] Oh Y., Ahn C.-B., Yoon N.Y., Nam K.H., Kim Y.-K., Je J.-Y. (2018). Protective effect of enzymatic hydrolysates from seahorse (*Hippocampus abdominalis*) against H_2_O_2_-mediated human umbilical vein endothelial cell injury. Biomed. Pharmacother..

[16] Suleria H.A.R., Gobe G., Masci P., Osborne S.A. (2016). Marine bioactive compounds and health promoting perspectives; innovation pathways for drug discovery. Trends Food Sci. Technol..

[17] Harnedy P.A., FitzGerald R.J. (2012). Bioactive peptides from marine processing waste and shellfish: A review. J. Funct. Foods.

[18] Ahn C.B., Je J.Y. (2019). Bone health-promoting bioactive peptides. J. Food Biochem..

[19] Yang X.-R., Qiu Y.-T., Zhao Y.-Q., Chi C.-F., Wang B. (2019). Purification and characterization of antioxidant peptides derived from protein hydrolysate of the marine bivalve mollusk *Tergillarca granosa*. Mar. Drugs.

[20] Kim S.S., Ahn C.-B., Moon S.W., Je J.-Y. (2018). Purification and antioxidant activities of peptides from sea squirt (*Halocynthia roretzi*) protein hydrolysates using pepsin hydrolysis. Food Biosci..

[21] Jin J.-E., Ahn C.-B., Je J.-Y. (2018). Purification and characterization of antioxidant peptides from enzymatically hydrolyzed ark shell (*Scapharca subcrenata*). Process Biochem..

[22] Park S.Y., Je J.-Y., Kang N., Han E.J., Um J.H., Jeon Y.-J., Ahn G., Ahn C.-B. (2017). Antihypertensive effects of Ile–Pro–Ile–Lys from krill (*Euphausia superba*) protein hydrolysates: Purification, identification and in vivo evaluation in spontaneously hypertensive rats. Eur. Food Res. Technol..

[23] Park S.Y., Kim Y.-S., Ahn C.-B., Je J.-Y. (2016). Partial purification and identification of three antioxidant peptides with hepatoprotective effects from blue mussel (*Mytilus edulis*) hydrolysate by peptic hydrolysis. J. Funct. Foods.

[24] Loboda A., Damulewicz M., Pyza E., Jozkowicz A., Dulak J. (2016). Role of Nrf2/HO-1 system in development, oxidative stress response and diseases: An evolutionarily conserved mechanism. Cell. Mol. Life Sci..

[25] Redza-Dutordoir M., Averill-Bates D.A. (2016). Activation of apoptosis signalling pathways by reactive oxygen species. Biochim. Biophys. Acta (BBA) Mol. Cell Res..

[26] Di Marzo N., Chisci E., Giovannoni R. (2018). The Role of Hydrogen Peroxide in Redox-Dependent Signaling: Homeostatic and Pathological Responses in Mammalian Cells. Cells.

[27] Fasanaro P., Magenta A., Zaccagnini G., Cicchillitti L., Fucile S., Eusebi F., Biglioli P., Capogrossi M.C., Martelli F. (2006). Cyclin D1 degradation enhances endothelial cell survival upon oxidative stress. FASEB J..

[28] Liu H.-T., He J.-L., Li W.-M., Yang Z., Wang Y.-X., Bai X.-F., Yu C., Du Y.-G. (2010). Chitosan oligosaccharides protect human umbilical vein endothelial cells from hydrogen peroxide-induced apoptosis. Carbohydr. Polym..

[29] Oh Y., Ahn C.B., Nam K.H., Kim Y.K., Yoon N.Y., Je J.Y. (2019). Amino Acid Composition, Antioxidant, and Cytoprotective Effect of Blue Mussel (*Mytilus edulis*) Hydrolysate through the Inhibition of Caspase-3 Activation in Oxidative Stress-Mediated Endothelial Cell Injury. Mar. Drugs.

[30] Saji N., Francis N., Blanchard C.L., Schwarz L.J., Santhakumar A.B. (2019). Rice Bran Phenolic Compounds Regulate Genes Associated with Antioxidant and Anti-Inflammatory Activity in Human Umbilical Vein Endothelial Cells with Induced Oxidative Stress. Int. J. Mol. Sci..

[31] Drummond G.S., Baum J., Greenberg M., Lewis D., Abraham N.G. (2019). HO-1 overexpression and underexpression: Clinical implications. Arch. Biochem. Biophys..

[32] Chiang S.K., Chen S.E., Chang L.C. (2019). A Dual Role of Heme Oxygenase-1 in Cancer Cells. Int. J. Mol. Sci..

[33] Zhan Y., Kim S., Izumi Y., Izumiya Y., Nakao T., Miyazaki H., Iwao H. (2003). Role of JNK, p38, and ERK in platelet-derived growth factor-induced vascular proliferation, migration, and gene expression. Arter. Thromb. Vasc. Biol..

[34] Joo Choi R., Cheng M.S., Shik Kim Y. (2014). Desoxyrhapontigenin up-regulates Nrf2-mediated heme oxygenase-1 expression in macrophages and inflammatory lung injury. Redox Biol..

[35] Nguyen T., Nioi P., Pickett C.B. (2009). The Nrf2-antioxidant response element signaling pathway and its activation by oxidative stress. J. Biol. Chem..

[36] Zhang Z., Jiang S., Zeng Y., He K., Luo Y., Yu F. (2020). Antioxidant peptides from *Mytilus Coruscus* on H_2_O_2_-induced human umbilical vein endothelial cell stress. Food Biosci..

[37] Je J.-Y., Lee D.-B. (2015). Nelumbo nucifera leaves protect hydrogen peroxide-induced hepatic damage via antioxidant enzymes and HO-1/Nrf2 activation. Food Funct..

[38] Moritani C., Kawakami K., Shimoda H., Hatanak T., Suzaki E., Tsuboi S. (2020). Protective effects of rice peptide Oryza Peptide-P60 against oxidative injury through activation of Nrf2 signaling pathway in vitro and in vivo. ACS Omega.

[39] Wang J., Wu T., Fang L., Liu C., Liu X., Li H., Shi J., Li M., Min W. (2020). Peptides from walnut (*Juglans mandshurica* Maxim.) protect hepatic HepG2 cells from high glucose-induced insulin resistance and oxidative stress. Food Funct..

[40] Cai S.-Y., Wang Y.-M., Zhao Y.-Q., Chi C.-F., Wang B. (2019). Cytoprotective effect of antioxidant pentapeptides from the protein hydrolysate of swim bladders of miiuy croaker (*Miichthys miiuy*) against H_2_O_2_-mediated human umbilical vein endothelial cell (HUVEC) injury. Int. J. Mol. Sci..

[41] Liang Y., Lin Q., Huang P., Wang Y., Li J., Zhang L., Cao J. (2018). Rice bioactive peptide binding with TLR4 to overcome H_2_O_2_-induced injury in human umbilical vein endothelial cells through NF-κB signaling. J. Agric. Food Chem..

[42] Ahn C.-B., Shin T.-S., Seo H.K., Je J.-Y. (2012). Phenolic composition and antioxidant effect of aqueous extract of *Arisaema cum* Bile, the oriental herb medicine, in human fibroblast cells. Immunopharmacol. Immunotoxicol..

