# Supplementary material for: Cytoprotective Role of Edible Seahorse (Hippocampus abdominalis)-Derived Peptides in H2O2-Induced Oxidative Stress in Human Umbilical Vein Endothelial Cells
Source: Mar Drugs. 2021 Feb 3;19(2):86. doi: 10.3390/md19020086 (PMC7913330; doi:10.3390/md19020086)
Supplement: Supplementary file 1 [file marinedrugs-19-00086-s001.pdf]

marine drugs

MDPI

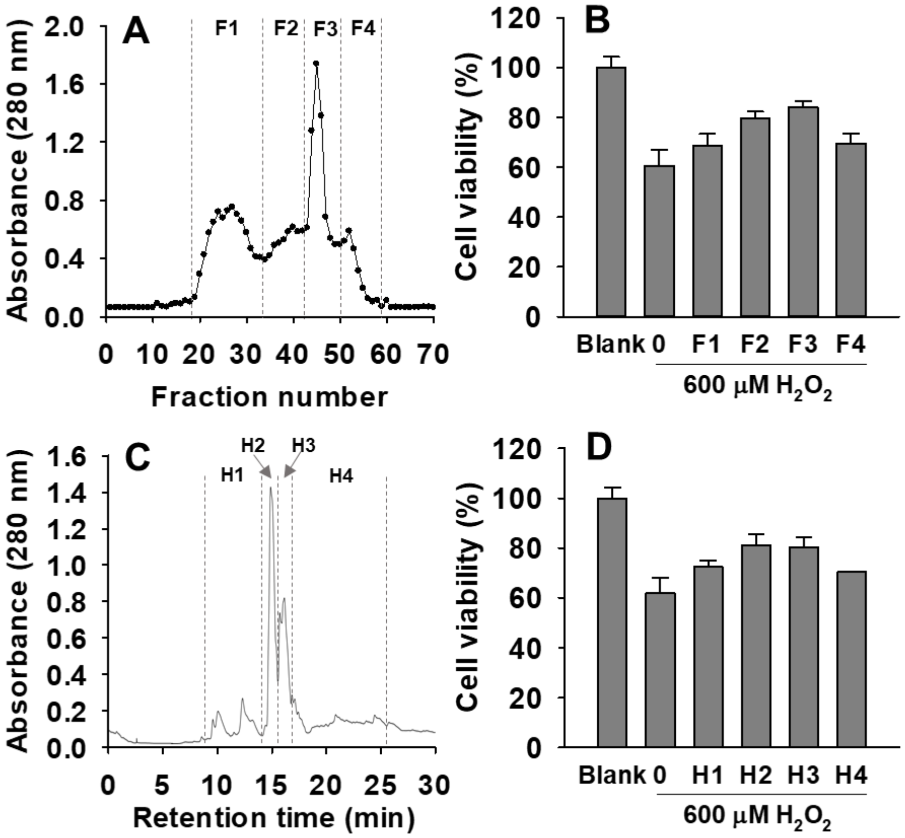

Supplementary Figure 1. Purification of cytoprotective peptides. (A) Gel filtration chromatogram obtained from Sephadex G-25, (B) Cytoprotective effect of gel filtration fractions, (C) HPLC chromatogram of the active fraction of gel filtration chromatography, and (D) Cytoprotective effect of HPLC fractions. Separation conditions were described in the Section 4.3. Cell viability assay were performed at 0.5 mg/mL (B) and 0.25 mg/mL (D). Values are presented as means ± SD.
